# How women’s empowerment influences fertility-related outcomes and contraceptive practices: A cross-sectional study in Mozambique

**DOI:** 10.1371/journal.pgph.0000670

**Published:** 2022-09-12

**Authors:** Sofia Castro Lopes, Deborah Constant, Sílvia Fraga, Jane Harries

**Affiliations:** 1 Division of Social and Behavioural Sciences, School of Public Health and Family Medicine, University of Cape Town, Cape Town, South Africa; 2 EPIUnit–Institute of Public Health, University of Porto, Porto, Portugal; University of Southern Denmark: Syddansk Universitet, DENMARK

## Abstract

Women’s empowerment could be a crucial step for tackling gender inequality and improve women’s reproductive health and rights in Mozambique. This study aims to examine how different domains of women’s empowerment influence fertility-related outcomes and contraceptive practices in Mozambique. We used the 2015 Demographic Health Survey (DHS) conducted in Mozambique from which a sample of 2072 women aged 15 to 49 years were selected and included in this analysis. A principal component analysis was performed, and the components retained were identified as the domains of empowerment. These were: Beliefs about violence against women, Decision-making, and Control over sexuality and safe sex. A multinomial logistic regression was run to estimate the association between levels of empowerment for each domain and the study outcomes. Crude and adjusted odds ratio (OR) were calculated, with 95% confidence intervals (95% CI). Beliefs about violence against women and Control over sexuality and safe sex were positively associated with having 1 to 4 children. Control over sexuality and safe sex also increased likelihood of women wanting to space childbearing over more than 2 years. Decision-making increased the odds of women not wanting more children. Middle to high empowerment levels for Control over sexuality and safe sex also increased the chances of using any type of contraceptive method and using it for longer periods. All domains, from the middle to high levels of empowerment, decreased the chances of women not wanting to use contraception. Our study confirmed the multidimensional nature of empowerment showing that each domain had a different effect over specific fertility and contraceptive outcomes and reinforced the importance of a domain approach for estimating and understanding empowerment. It also revealed the critical role of Control over sexuality and safe sex domain for improving women’s ability to decide over fertility and contraceptive practices in Mozambique.

## Introduction

In 1994, during the International Conference on Population and Development (ICPD) in Cairo, the right of women to freely decide on their reproductive lives without discrimination was recognised by nations around the world [1]. More recently, the Sustainable Development Goal (SDG) 5 reiterated that women’s empowerment is a priority for improving family planning and reproductive health outcomes and for tackling gender inequality [2–4]. Empowerment enables women to decide and act on their decisions which is key for informed decision-making in accessing family planning services and using of modern methods of contraception [5].

Women’s empowerment as defined by Kabeer includes “the expansion of people’s ability to make strategic life choices in a context where this ability was previously denied” [6]. The process of empowerment is individual, involving building a critical consciousness of women’s rights and changing unequal gender power relations [7]. When women understand that they can aspire to a different life and that it is their right to decide, their ability to make strategic life choices is enhanced [8]. They are enabled to make decisions, including about their sexual and reproductive lives [1], such as deciding freely the number, spacing and timing of childbearing, which then influences other life changing decisions and opportunities including to have a job, a career, political participation, among others.

Resources or pre-conditions are an essential component for the process of women’s empowerment [6]. This includes not only the access to material resources such as education or financial resources but also human and social capital [6]. Recent studies have expanded on individual, and structural factors that can influence the process of empowerment [9–11]. For example, women’s age, where they live or participating in women’s associations (networking with other women) play an important role in how empowerment unfolds [10].

Evidence has shown the influencing role of women’s empowerment in reproductive health behaviours. Overall empowerment has been associated with women’s desire in having a smaller number of children, using modern methods of contraception, having higher levels of met need for contraception and having better spousal communication [4, 12, 13]. However, the results available in the literature are inconsistent across countries and different associations have been found between different dimensions of empowerment and reproductive outcomes [13, 14]. Often this is related to the conceptualization and operationalization of empowerment [13, 14] Studies use different definitions of empowerment, not always fully based in the evidence and theory available [15]. While empowerment’s multidimensional nature has been recognised, overall empowerment is still used to quantitatively measure it. Empowerment is a process that occurs in different spheres of a woman’s life and through different pathways [16] and recent evidence supports the use of specific domains of empowerment in its operationalisation and measurement. For example, measuring reproductive empowerment when considering women’s ability to make decisions on sexual and reproductive health [5].

As empowerment became a mainstream concept and part of development strategies, a shift in focus occurred, which also contributed to different approaches and definitions [8]. Great emphasis was put into women’s access to materials and resources, not considering the internal processes of questioning and change that are encompassed in the empowerment process [8]. This has created fragilities in understanding of empowerment. Furthermore, the attempts to capture it quantitatively was shown to be challenging. The use of Demographic and Health Survey (DHS) as the main source of data offers many advantages, such as standardization and comparability, however the availability of indicators and depth of what it captures is limited [7, 13], impacting on how empowerment is operationalised and possible conclusions. Mozambique, like other sub-Saharan countries, has experienced a decline in fertility levels only in the last decade. The total fertility rate at national level was 5.5 in 2003, 5.9 in 2011 [17], 5.2 in 2016 and 4.8 in 2019 [18, 19]. The prevalence of the use of modern contraceptives remains low. From 2003 to 2011, contraceptive prevalence decreased from 17% to 12% [17], followed by a steady increase to 25% and to 35% in 2015 and in 2019, respectively [20]. The change in the trend of contraceptive prevalence may result from a strengthened commitment from the government not only to improve access and supply to contraceptives but also to increase the educational levels of girls and women, improve their knowledge on contraception and reduce gender-based violence [21, 22].

Important disparities between urban and rural areas remain, with rural areas presenting small changes in the high fertility rate and low contraceptive use patterns [23]. Also, studies on fertility transition in various developing countries show that despite the presence of triggering factors for lowering fertility rates, such as economic and social development, decrease in mortality, desire for smaller families and awareness that childbearing can be planned [24, 25], this may not result in a decrease of fertility if women are not able to control their fertility through the use of modern contraception and according to their preferences [26, 27].

In patriarchal societies like Mozambique, men are entitled to exert control over women, particularly with respect to the number of children, spacing of pregnancies and use of contraceptives [26, 28]. This is based on deep-rooted sociocultural practices and traditions, where large families are valued and women’s social recognition (and value) is tied to how many children she can bear [26]. Despite the efforts of the government in implementing and expanding family planning programmes focussing on the free supply of modern methods of contraception and sensitization and knowledge campaigns about modern contraception [26], evidence has shown that these actions may not be enough to enable women to use them if not empowered or in control of their reproductive health [29].

As gender inequality remains one of the main barriers for women’s ability to use family planning methods and make reproductive choices in Mozambique [22, 28], women’s empowerment could contribute towards tackling gender inequality and improve women’s reproductive health and rights. Enabling women to make decisions about their bodies and reproductive lives, based on information and free of coercion, contributes largely to the fulfilment of their human, sexual and reproductive rights, to living up to their full capabilities, and to better health outcomes [30, 31].

Understanding how women’s empowerment work and which reproductive outcomes are more influenced by it in the Mozambican context, could support and inform the design of health and gender strategies. This study aims to examine how different domains of women’s empowerment influence fertility related outcomes and contraceptive practices in Mozambique.

## Methods

### Data source

We used the 2015 Demographic Health Survey (DHS) conducted in Mozambique which included indicators about women’s empowerment, fertility-related outcomes, and contraceptive practices [23]. The DHS is part of a USAID program that supports countries to monitor and evaluate their demographic and health parameters at national and subnational levels [32] and the datasets can be assessed online upon registration.

The 2015 DHS was a population-based survey, involving the 11 provinces of Mozambique, which collected data from women and men, aged 15 to 59. In total, 7749 women and 5283 men were interviewed from 7129 households included in the survey.

The survey’s section about empowerment was applied to married women only, and the section about domestic violence to a sub-sample of female participants. The criteria of participants inclusion in this analysis were age (15 to 49 years) and those who answered all sections of the survey, including the section about empowerment, and the section on domestic violence. A total of 2072 women of reproductive age (15 to 49 years) were included in this analysis. Those excluded (unmarried/unpartnered women [n = 4874]), when compared to participants included, had fewer children, different intention to childbearing, and a higher proportion of contraceptive met- or no need as they were not sexually active.

### Variables

#### Outcomes

*1*. *Fertility-related outcomes*. Three indicators characterising women’s fertility were selected from the DHS Mozambique 2015. This included the number of children ever born, categorised in ‘0’, ‘1–4’, ‘5 or more’, based on the total fertility rate of 4.8 in 2019 [19]. Also, this was based on the theoretically plausibility that women with no children or with more than expected average may have different levels of empowerment. Time interval between the last 2 births in the 6 years prior to the survey was also used as an outcome. The categories defined for this variable were ‘Less than 2 years’, ‘2 to 6 years’, and ‘1 or less children born in the previous 6 years’. It was decided not to exclude women the latter group of women, after confirming that no significant changes existed after exclusion. The last fertility outcome included in this study was intention for childbearing, defined as women’s desire of wanting to and when to have a child in the future. This outcome was categorized in the following way: ‘Less than 2 years’, ‘2 years or more’, ‘Undecided’, and ‘Does not want’.

*2*. *Contraceptive practices*. From the DHS 2015 we selected three outcomes that describe contraceptive practices of women, namely: Current use of contraceptives (No use, Modern, and Traditional), Length of use (2 years or less, More than 2 years, and No use), and Met need for contraception (Unmet need, Met need, No want—despite being sexually active; and, No need–those not sexually active, infertile or menopause).

### Independent variables

#### Empowerment domains

We identified empowerment domains in a previous study [10]. In brief, the selection process of empowerment consisted of the identification of the relevant questions related to empowerment from the DHS 2015 survey for Mozambique. The relevancy was assessed by current evidence available and theoretical plausibility as well as the definition of empowerment used in the study. The included indicators related to women’s decision-making within the household (Who usually decides on visits to family and friends, on large purchases, and women’s health care), women’s justified beating (if wife goes out without telling husband, if neglects the children, if argues with husband, if refuses to have sex, if burns the food), and decisions about sexual intercourse (wife an ask husband to use a condom, to use a condom if he has a sexually transmitted disease, can refuse sex). Each question was coded into a 3-point scale (values of -1, 0, 1) and the highest value was given to categories considered to indicate greater level of empowerment [7, 10]. For women’s justified beating, the answers ‘Not justified`were coded 1, while ‘Justified ‘-1, and ‘Don’t know’ as 0.

A principal component analysis (PCA) was performed [7, 33, 34] which allowed the assessment of how the selected empowerment indicators cluster and how much each contributes to a specific component [7]. The components with an eigen value above 1 were considered significant and therefore were retained. The scree plot of the PCA is included in (S1 Fig) and the factor loadings of the retained components are shown in S2 Table of the article Castro Lopes et al, 2021 [10]. The Kaiser Meyer-Olkin test value was 0.75 which confirmed the sampling adequacy for PCA [10].

Based on the indicators clustering and contribution to each retained component, we identified and named three domains of empowerment: ‘Beliefs about violence against women’; ‘Decision-making’, and; ‘Control over sexuality and safe sex’ [10]. The three retained components explained 60% of the total variance in the data set [10]. Further detail on the PCA estimates can be obtained in Castro Lopes et al (2021) [10].

The factors’ scores of the PCA for each indicator were used to estimate a domain-specific index. Given the distribution of the index of each domain of empowerment, and the need to understand if the study outcomes, vary across levels of empowerment, we stratified each domain of empowerment into terciles. The first tercile represented women with low levels of empowerment, the second tercile, women with middle levels, and the third, those with high levels of empowerment.

### Covariates

#### Socio-economic, demographic and behavioural characteristics

The key role of women’s sociodemographic and economic characteristics for the empowerment process has been widely recognised [35]. Some of these characteristics are considered essential pre-conditions or resources for the process of empowerment to take place as they enable women’s decision-making and agency [6, 11]. These encompass education, employment, financial resources, among others [6, 16]. Empowerment levels are also expected to change throughout life, with age [35]. Therefore, the following covariates were included in the analysis: women’s age (Less or equal to 19, 20–29, 30–39, 40–49 years), education (No education, Primary - 1st to 7th grade, secondary and above - 8th and above), and current employment situation reported by women (Working, Not working). Wealth index was also used, a composite measure estimated by the DHS, based the ownership of assets [23]. The index was recoded into quintiles: poorest, poor, middle, rich, richest.

Furthermore, the number of living children (0, 1–4, 5 or more) was considered a covariate as it can influence or change the fertility preferences of women, including the intention or wish of future childbearing.

Provinces were combined into regions, following the official aggregation of provinces by the Mozambique government into South, Centre, and North regions [36]. Urban and rural residency areas were also considered as evidence suggests the existence of important differences [10].

Behavioural characteristics, including women’s exposure to controlling behaviours from their partners (No control; At least one type of control) and intimate partner violence (IPV—Yes, No) experienced in the past 12 months [23] were included in the analysis as these are considered main barriers to women’s empowerment processes [37, 38].

### Data analysis

A descriptive analysis of the outcome variables, empowerment domains and the socio-economic, demographic and behavioural characteristics was performed. Proportions were compared using the Chi-square test (level of significance 5%).

A multinomial logistic regression was run to estimate the association between levels of empowerment for each domain and the fertility-related outcomes and the contraceptive practices. Crude and adjusted odds ratio (OR) were calculated, with 95% confidence intervals (95% CI). The final models were adjusted for both statistically and theoretical relevant socio-economic, demographic, and behavioural characteristics. These included number of live children, age, education, region, and wealth quintile. Hosmer–Lemeshow goodness-of-fit test for multinomial logistic regression models was used to test the fit of the models [39].

We used the STROBE cross sectional checklist when writing the results section [40].

### Ethics statement

This study is a secondary data analysis of DHS data [32]. The DHS program secured ethical clearance and participant informed consent [23].

## Results

### Descriptive results

Table 1 described women’s Socio-economic, demographic, and behavioural characteristics. Most women had a primary level of education, were 20 to 29 years old, unemployed, belonged to the middle to richest wealth quintiles, lived in rural areas and Central region. More than 40% of women reported being exposed to controlling behaviours from their partner and 23% to IPV (Table 1). Similar characteristics were found among the DHS female participants, except for age, where our sample was slightly older. This could be related to the inclusion of only married or partnered women.

**Table 1:** Women’s socio–economic, demographic, and behavioural characteristics.

Women’s sociodemographic, economic and behavioural characteristics	
**Age**	
<19	164 (7.4)
20–29	849 (41.0)
30–39	626 (30.2)
40–49	433 (20.9)
**Education** ^**1**^	
No education	604 (29.2)
Primary (1^st^ to 7^th^ grade)	1086 (52.4)
Secondary and above (8^th^ and above)	382 (18.4)
**Number of living children**	
0	161 (7.8)
1–4	1376 (66.4)
5 or more	535 (25.8)
**Currently employed**	
Yes	912 (44.0)
**Wealth index**	
Poorest	337 (16.3)
Poorer	381 (18.4)
Middle	430 (20.8)
Richer	475 (22.9)
Richest	449 (21.7)
**Regions**	
North region	602 (29.1)
Central region	843 (40.7)
South region	627 (30.3)
**Urban vs rural residency**	
Rural	1321 (63.8)
**Partner/Husband controlling behavior**	
No control	1191 (57.5)
At least one type	881 (42.5)
**IPV exposure**	
No	1596 (77.2)

^1^ Based on the previous education system organization. System changed in 2018.

Table 2 describes the outcome variables and the empowerment domains. More than 60% of women had 1 to 4 children (average births was 3.7 [SD ±2.6]) and 63% had 1 or less children in the 6 years prior to the survey. Most women wished not to have more children or to wait more than 2 years to have another child. However, the contraceptive practices showed low uptake of modern contraceptives (approximately 30%), with 20% of women using for more than 2 years. Interestingly, 30% of women reported unmet need for contraception while 30% did not want to use contraception. Most women had high level of empowerment for ‘Decision-making’ and middle levels of empowerment for ‘Beliefs about violence against women’ and, ‘Control over sexuality and safe sex’ domains of empowerment (Table 2). S1 Table describes the outcome variables by selected socio-economic, demographic, and behavioural characteristics of women (see S1 Table).

**Table 2:** Description of outcome variables and the empowerment domains.

** **Outcomes** **	**N = 2072**		
**Fertility-related**		**Contraceptive practices**	
**Number of children ever born**		**Current use of contraceptive methods**	
0	122 (5.9)	No method	1441 (69.6)
1–4	1265 (61.1)	Modern method	604 (29.2)
5 or more	685 (33.0)	Traditional method	27 (1.3)
**Time interval between the last 2 births** ^**1**^		**Length of use of contraceptives**	
Less than 2 years	141 (6.8)	No use	1450 (70.0)
2 to 6 years	630 (30.4)	2 years or less	430 (20.8)
1 or less children born in the previous 6 years	1301 (62.8)	More than 2 years	192 (9.3)
**Intention for childbearing**		**Need for contraceptives**	
Less than 2 years	413 (20.0)	Met need	461 (22.3)
2 years or more	710 (34.4)	Unmet need	631 (30.5)
Undecided	222 (10.8)	No need (no want)	611 (29.5)
Does not want	718 (34.8)	No physiological need^2^	366 (17.7)
**Domains of empowerment, terciles, N (%)**			
**Beliefs about violence against women**			
Low	652 (31.5)		
Middle	743 (35.9)		
High	677 (32.7)		
**Decision-making**			
Low	653 (31.5)		
Middle	675 (32.6)		
High	744 (35.9)		
**Control over sexuality and safe sex**			
Low	715 (34.5)		
Middle	786 (37.9)		
High	571 (27.6)		

^1^ The difference in months between the two most recent births in the prior 6 years to the DHS survey. ^2^ Women who are not sexually active or are infertile.

Table 3 describes the association between the fertility-related outcomes and contraceptive practices by empowerment levels for each domain. Women with 5 or more children had lower levels of empowerment for ‘Beliefs about violence against women’ and ‘Control over sexuality’, while not wanting more children was associated with high levels of ’Decision-making’. No children was also associated with lower levels of ´Control over sexuality´. For contraceptive practices, the use of modern contraceptives was associated with women with middle level of empowerment for ‘Beliefs about violence against women’ and ‘Control over sexuality and sex’ and high level of ´Decision-making´. The use of traditional contraceptives was also associated with high levels of empowerment for ´Decision-making´ and ‘Beliefs about violence against women’. Longer periods of use of contraceptives and met need for contraception were associated with middle and higher levels of ‘Control over sexuality and sex’ and ´Decision-making´, respectively.

**Table 3:** Description of fertility–related outcomes and contraceptive practices by women’s empowerment domains in Mozambique.

**Fertility-related outcomes**													
	**Number of children ever born**				**Time interval between the last 2 births**				**Intention for childbearing**			
	**0**	**1–4**	**5 or more**	**p-value**	**Less than 2 years**	**2 to 6 years**	**1 or less children born** ^**2**^	**p-value**	**Less than 2 years**	**2 years or more**	**Undecided**	**No want**	**p-value**
**Beliefs about violence** ^**1**^													
Low	39 (31.0)	362 (28.6)	247 (36.1)	0.012	36 (29.8)	213 (32.8)	403 (30.9)	0.933	123 (29.8)	225 (31.6)	75 (33.9)	223 (31.1)	0.246
Middle	47 (38.5)	561 (44.6)	278 (40.6)		45 (37.2)	229 (35.2)	469 (36.1)		190 (46.0)	299 (42.1)	101 (45.5)	290 (40.4)	
High	36 (29.5)	391 (27.0)	160 (23.4)		40 (33.1)	208 (32.0)	429 (33.0)		100 (24.2)	186 (26.2)	46 (20.7)	205 (28.5)	
**Decision-making**													
Low	41 (33.6)	390 (30.8)	222 (32.4)	0.800	41 (33.8)	229 (35.2)	383 (29.4)	0.061	139 (33.7)	254 (35.8)	74 (33.3)	182 (25.4)	< .001
Middle	39 (31.9)	424 (33.5)	212 (31.0)		40 (33.1)	211 (32.5)	424 (32.6)		143 (34.6)	211 (29.7)	83 (37.4)	235 (32.7)	
High	42 (34.4)	451 (35.7)	251 (36.6)		40 (33.1)	210 (32.3)	494 (38.0)		131 (31.7)	245 (34.5)	65 (29.3)	301 (41.9)	
**Control over sexuality and safe sex**													
Low	50 (41.0)	382 (30.2)	283 (41.3)	< .001	41 (33.9)	238 (36.6)	436 (33.5)	0.344	157 (38.0)	225 (31.7)	87 (39.2)	242 (33.7)	0.063
Middle	42 (34.4)	485 (38.3)	259 (37.8)		50 (41.3)	226 (34.8)	510 (39.2)		162 (39.3)	267 (37.6)	81 (36.5)	273 (38.0)	
High	30 (24.6)	398 (31.5)	143 (20.9)		30 (24.8)	186 (28.6)	355 (27.3)		94 (22.8)	218 (30.7)	54 (24.3)	203 (28.3)	
	**Contraceptive practices**												
	**Current use of contraceptive methods**				**Length of use of contraceptives**				**Need for contraceptives**			
	**No use**	**Modern**	**Traditional**	**p-value**	**No use**	**2 years or less**	**More than 2 years**	**p-value**	**Unmet need**	**Met need**	**No want**	**No need**	**p-value**
**Beliefs about violence** ^**1**^													
Low	452 (31.4)	195 (32.3)	5 (18.5)	< .001	455 (31.4)	138 (32.1)	59 (30.7)	0.097	131 (28.4)	200 (31.7)	206 (33.7)	115 (31.4)	0.023
Middle	495 (34.4)	243 (40.2)	5 (18.5)		499 (34.4)	162 (37.7)	82 (42.7)		179 (38.8)	248 (39.3)	187 (30.6)	129 (35.3)	
High	494 (34.3)	166 (27.5)	17 (63.0)		496 (34.2)	130 (30.2)	51 (26.6)		151 (32.8)	183 (29.0)	218 (35.7)	122 (33.3)	
**Decision-making**													
Low	476 (33.0)	167 (27.7)	10 (37.0)	0.002	479 (33.0)	131 (30.5)	43 (22.4)	< .001	147 (31.9)	177 (28.1)	228 (37.3)	100 (27.3)	< .001
Middle	484 (33.6)	187 (31.0)	4 (14.8)		488 (33.7)	134 (31.2)	53 (27.6)		154 (33.4)	191 (30.3)	179 (29.3)	149 (40.7)	
High	481 (33.4)	250 (41.4)	13 (48.2)		483 (33.3)	165 (38.4)	96 (50.0)		160 (34.7)	263 (41.7)	204 (334)	117 (32.0)	
**Control over sexuality and safe sex**													
Low	566 (39.3)	144 (23.8)	5 (18.5)	< .001	571 (39.4)	107 (24.9)	37 (19.3)	< .001	166 (36.0)	149 (23.6)	250 (40.9)	147 (40.2)	< .001
Middle	533 (37.0)	236 (39.1)	17 (63.0)		534 (36.8)	163 (37.9)	89 (46.4)		167 (36.2)	253 (40.1)	224 (36.7)	142 (38.8)	
High	342 (23.7)	224 (37.1)	5 (18.5)		345 (23.8)	160 (37.2)	66 (34.4)		128 (27.8)	229 (36.3)	137 (22.4)	77 (21.0)	

^**1**^ Beliefs about violence against women; ^2^ in the last 6 years prior to the survey.

### Relationship of empowerment with fertility-related outcomes and contraceptive practices

**Fig 1:**
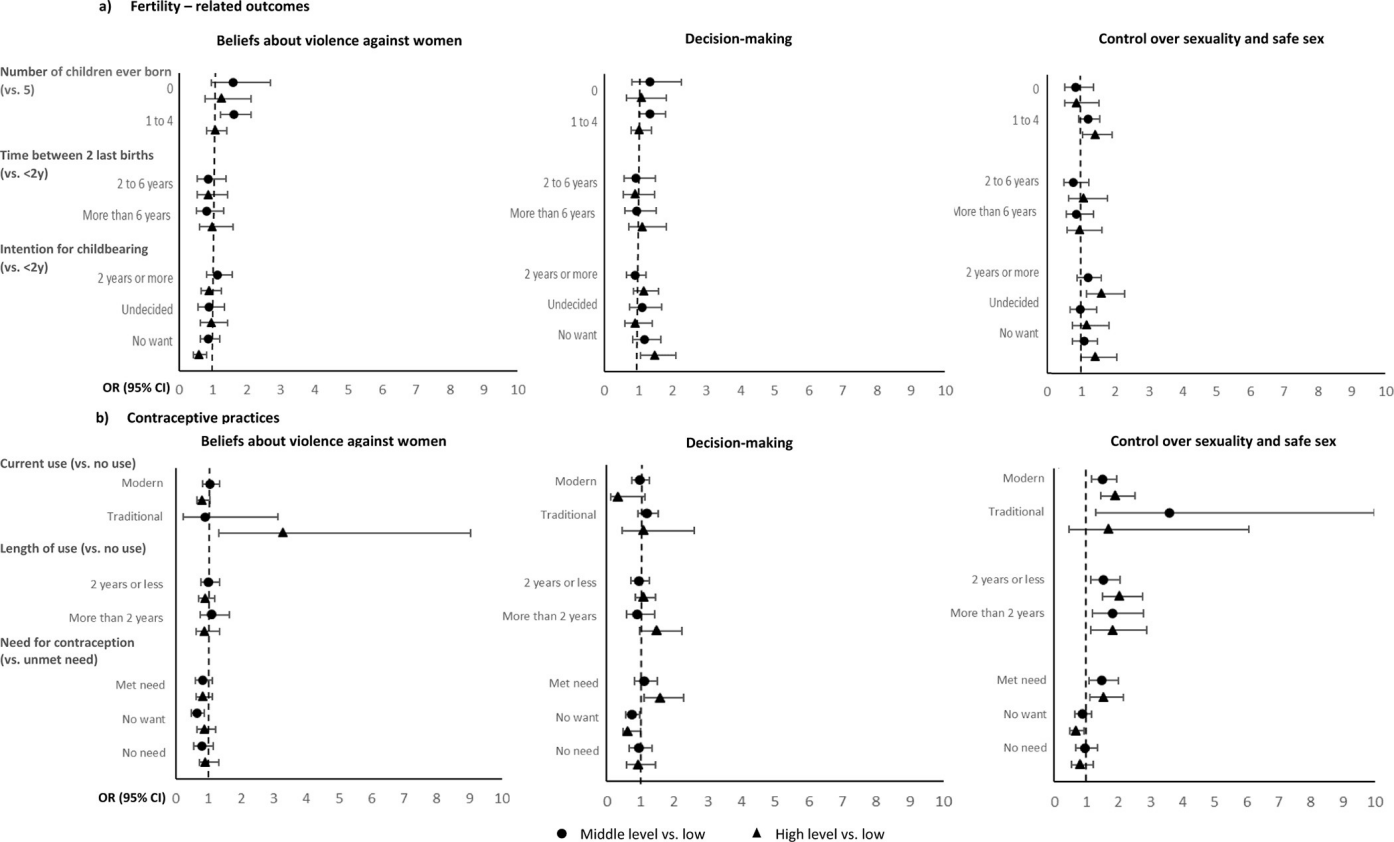
Adjusted odds ratio with 95% CI from multinomial regression analysis to identify the association between the domains of women’s empowerment and a) Fertility related outcomes (adjusted for number of live children, age, education and region), and; b) contraceptive practice (adjusted for number of live children, age, education).

Fig 1 presents the results from the multinomial adjusted logistic regression for fertility-related outcomes and contraceptive practices (crude and adjusted OR provided in S2 Table). Fig 1A) shows that middle of empowerment for the domain ‘Believes about violence against women’ and high level of empowerment for ‘Control over sexuality and safe sex’ were positively associated with having 1 to 4 children when compared with women with low levels of empowerment. Having high empowerment for ‘Control over sexuality’ also increased likelihood of women wanting to space childbearing for more than 2 years when compared with women with low levels of empowerment. Women with high ‘Decision-making’ power had increased odds of not wanting more children compared with those with low level of empowerment and who want in less than 2 years and. Being empowered for ‘Beliefs about violence’ decreased the likelihood of women not wanting children.

Fig 1B) shows that women with high levels of empowerment for ‘Believes about violence against women’ had increased likelihood of using currently traditional methods of contraception, while high empowerment for ‘Control over sexuality and safe sex’ increased the odds of using modern contraceptives when compared to women in the lowest level of empowerment. Middle to high empowerment levels for ‘Control over sexuality and safe sex’ also increased the chances of using any type of contraceptive methods, traditional or modern, and using it for longer periods (less or more than 2 years). Having high empowerment for the domains ‘Control over sexuality and safe sex’ and ‘Decision-making’ was positively associated with contraceptive met need when compared to low levels of empowerment in these domains. All domains, from middle to high levels of empowerment, decreased the likelihood of women not wanting to use contraception.

## Discussion

Our results suggest that women’s empowerment influences women’s contraceptive practices and fertility outcomes. However, each domain of empowerment influences differently the various fertility and contraceptive outcomes. In particular, the domains of ‘Control over sexuality and safe sex’, and of ‘Decision-making’ seem to be important for the context of Mozambique. This highlights the importance of considering the multidimensionality of empowerment and its relationship with specific areas of a woman’s life within a particular social context. Recent efforts have been made to conceptualize specific areas of empowerment, such as reproductive empowerment or empowerment in sexual and reproductive health, [35] and our findings support this approach. This challenges the use of the DHS data to quantify empowerment, which has often lead to ad hoc measures of overall empowerment, with weak conceptualization [15]. Expansion of the current indicators of empowerment from the DHS and other global sources of data is required, together with qualitative approaches to grasp context-specific aspects of the empowerment process and its influence in sexual and reproductive health.

In some African countries, women’s decision-making power in the household has been associated with their ability to make their own decisions in terms of reproductive choices, including desiring fewer children and in some countries with the use of contraceptives [41]. Our study showed that high levels of decision-making power was positively associated with not wanting more children and met the need for contraception. This suggests that when women in Mozambique decide limiting childbearing, they are more likely to have this need satisfied. The relationship between ‘Decision-making’ and current use of contraception, specifically, has not been established in the literature with studies reporting both null or positive effect especially after adjusting for community characteristics [13] which supports our findings. This could be explained by the various ways of operationalising ‘Decision-making’ domain of empowerment, oftentimes informed by an ad hoc process of estimation [14]. Furthermore, one should note that the current use of contraception could be dependent on the availability and accessibility of contraceptive services, which do not necessarily dependent on women’s decision-making power levels [13].

Our study showed that ‘Control over sexuality and safe sex’ is a key empowerment domain for both women’s fertility decision and contraception practices, and this relationship is independent of women’s education and wealth. The ‘Control over sexuality and safe sex’ has been less explored in the literature as a domain of empowerment therefore evidence is scarce. A possible interpretation of our findings is that women’s perception and understanding of their right to sexual and reproductive health might be a key for both the process of empowerment as well as to the choices themselves [29, 42, 43]. Thus, it is the building critical consciousness of having rights and possibilities of women that are fundamental for the many decisions that unfold from there [42].

‘Beliefs about violence against women’ domain of empowerment seemed to play a less important role for the outcomes studied. Contrary to what has been shown in a multi-country study that included Mozambique [7], we did not find any association between high levels of empowerment for this domain and the current use of modern contraception. A possible reason for the different results is that current use of modern methods was computed as a binary outcome (using, not using) in the other study. However, we observed that empowered women in this domain, were more likely to use traditional methods of contraception. This could point to the strength of community believes and traditions in women’s choices [11] and also reinforces the multidimensionality of empowerment and how this might play an less important role for reproductive empowerment. Furthermore, women with middle level of empowerment for ‘Beliefs about violence against women’ had an increased chance of having less children than those with high level. Such observation could be related to experiencing an environment where violence against women is accepted and normalised [44], and women tolerate it more, but which could result in women wanting to have less children as a protection mechanism.

Women’s empowerment, in each of its domains, does not seem to influence the time interval between the last two births in Mozambique, with our results showing null associations. Similar results have been described in other studies in the African context and the explanation put forward suggests that large time intervals (average 35 months) [25] are often observed in these settings due to cultural practices and traditions which expose women to long abstinence periods postpartum [45]. This highlights the importance of assessing the utility and relevance of the selected outcomes for the context [13].

Our findings support the idea that empowerment influences fertility and contraceptive outcomes through different pathways and it offers an opportunity to hypothesize about possible routes through which empowerment works [41]. In our results we saw that ‘Decision-making’ increased the chances of a woman not wanting more children and having her contraceptive needs met, but this was not reflected in the likelihood of having smaller families or current contraceptive use. However, the domain ‘Control over sexuality and safe sex’, seem to also influence women’s ability to reduce the number of children ever born by prolonged use of modern contraceptives. A possible explanation for this different is that when women are empowered for ’Decision-making’ in the household they may still be prone to suffer the influence of the community and context; but when women are empowered on ‘Control over sexuality and safe sex’, they have incorporated the knowledge and understanding of the fundamental right to their bodies and participate in decisions that affect their lives [29], namely the ability to control the number of children they have.

Context-specific characteristics are also relevant for fertility-related outcomes. In Mozambique, like in other sub-Saharan countries, large families are still valued by society and shaped by cultural norms and traditions [28, 41, 45], where fertility is often associated with the woman’s status. Furthermore, evidence shows that women who reside in areas with high child and infant mortality levels, prefer and tend to have more children to compensate for the actual or anticipated loss of a child [46]. These contextual factors can modify the effect of women’s empowerment on reproductive outcomes.

### Strengths and limitations

This study used a population-based survey for Mozambique which allowed representativeness of the sample of the target population and generalizability of the findings. The large sample size ensured sufficient power for conducting the analysis. Despite these strengths, some limitations should be noted. Causality cannot be inferred due to the cross-sectional design of the DHS. A longitudinal approach would benefit the understanding of both how empowerment levels change as well as how it influences fertility outcomes and behaviors over time. Only partnered women were included in the analysis which should be considered in the interpretation of the findings. It is possible that other fertility outcomes would better describe the ability of women to make decisions and exercise choice. For example, the use of time intervals between births in the 6 years prior to the survey might not be the best variable to characterised fertility intentions. Also, the way this variable was categorised in this study might have reduce the validity and therefore compromise the interpretation of the findings. In the future, using women’s ideal number of children would help to better understand women’s ability/power of achieving this and through which pathways empowerment operates [4]. Similarly, the outcomes ever use of contraception or the intention of future use could provide an overview of contraception practices over time [13]. The empowerment domains were defined based on the available data in the DHS, there could be other indicators of relevance to describe women’s empowerment in Mozambique. Qualitative research involving Mozambican women could shed light on new and refine current empowerment domains and reproductive outcomes and may provide an explanation to understand why the domains influence differently each outcome.

## Conclusion

Our study confirmed the multidimensional nature of empowerment showing that each domain had a different effect over specific fertility and contraceptive outcomes. This reinforces the need of a domain approach to estimating and understanding empowerment. More importantly, our findings revealed the critical role of ‘Control over sexuality and safe sex’ domain for improving women’s ability to decide over fertility and contraceptive practices in Mozambique. This is important because it enables women’s basic fundamental rights, namely the right to have control over their bodies and the right to participate in decisions that concern their lives which in turn can enable their participation in society [7, 43]. We also highlighted the importance of context-specific factors for defining appropriate outcomes but also for a more accurate understanding of empowerment within the cultural and societal frame where it is being measured. While we believe this evidence could contribute to improving and refining existing family planning programmes in Mozambique, there is the need to continue this work closer to women lives and realities in Mozambique, so their voices can be included, and the nuances of the processes of empowerment can be captured.

## Supporting information

S1 Table A: Description of fertility-related outcomes by selected sociodemographic characteristics; and B: Description of contraceptive practices by selected sociodemographic characteristics.
(PDF)

S2 Table A: Crude and adjusted odds ratio (95% CI) from the multinomial logistic regression to estimate the association between the empowerment domains and fertility-related outcomes; and B: Crude and adjusted odds ratio (95% CI) from the multinomial logistic regression to estimate the association between the empowerment domains and contraceptive practices.
(PDF)

S1 Fig Scree plot of eigenvalues after PCA. (TIF)
