# Supplementary material for: How women’s empowerment influences fertility-related outcomes and contraceptive practices: A cross-sectional study in Mozambique
Source: PLOS Glob Public Health. 2022 Sep 12;2(9):e0000670. doi: 10.1371/journal.pgph.0000670 (PMC10021614; doi:10.1371/journal.pgph.0000670)
Supplement: S1 Table — A: Description of fertility-related outcomes by selected sociodemographic characteristics; and B: Description of contraceptive practices by selected sociodemographic characteristics. (PDF) [file pgph.0000670.s001.pdf]

S1 Table A: Description of the fertility-related outcomes by selected sociodemographic characteristics.

	Fertility-related outcomes												
	Number of children ever born				Time interval between the last 2 births				Intention for childbearing				
	0	1-4	5 years or more	p-value	Less than 2 years	2 to 6 years	More than 6 years	p-value	Less than 2 years	2 years or more	Undecided	No want	p-value
**Age**													
<19	35 (28.7)	129 (10.2)	0	<.001	16 (11.3)	19 (3.0)	129 (9.9)	<.001	40 (9.7)	101 (14.2)	15 (6.8)	8 (1.1)	<.001
20-29	58 (47.5)	708 (56.0)	83 (12.1)		69 (48.9)	338 (53.7)	442 (34.0)		192 (46.5)	415 (58.5)	86 (38.7)	154 (21.5)	
30-39	16 (13.1)	293 (23.2)	317 (46.3)		45 (31.9)	206 (32.7)	375 (28.8)		123 (29.8)	159 (22.4)	77 (34.7)	263 (36.6)	
40-49	13 (10.7)	135 (10.7)	285 (41.6)		11 (7.8)	67 (10.6)	355 (27.3)		58 (14.0)	35 (4.9)	44 (19.8)	293 (40.8)	
**Education[1]**													
No education	34 (27.9)	273 (21.6)	297 (43.4)	<.001	43 (30.5)	206 (32.7)	355 (27.3)	<.001	114 (27.6)	180 (25.4)	81 (36.5)	225 (31.3)	<.001
Primary	54 (44.3)	675 (53.4)	357 (52.1)		83 (58.9)	334 (53.0)	669 (51.4)		219 (53.0)	361 (50.9)	113 (50.9)	390 (54.3)	
Secondary and above	34 (27.9)	317 (25.1)	31 (4.5)		15 (10.6)	90 (14.3)	277 (21.3)		80 (19.4)	169 (23.8)	28 (12.6)	103 (14.4)	
**Currently employed**													
No	72 (59.0)	731 (57.8)	357 (52.1)	0.043	80 (56.7)	361 (57.3)	719 (55.3)	0.688	231 (55.9)	450 (63.4)	114 (51.4)	360 (50.1)	<.001
Yes	50 (41.0)	534 (42.1)	328 (47.9)		61 (43.3)	269 (42.7)	582 (44.7)		182 (44.1)	260 (36.6)	108 (48.7)	358 (49.9)	
**Live children**													
0	122 (100.0)	38 (3.0)	1 (0.15)	<.001	0	1 (0.2)	160 (12.3)	<.001	95 (23.0)	36 (5.1)	10 (4.5)	20 (2.8)	<.001
1-4	0	1227 (97.0)	149 (21.8)		80 (66.1)	424 (65.2)	872 (67.0)		281 (68.0)	574 (80.9)	148 (66.7)	369 (51.4)	
5 or more	0	0	535 (78.1)		41 (33.9)	225 (34.6)	269 (20.7)		37 (8.9)	100 (14.1)	64 (28.8)	329 (45.8)	
**Regions**													
North region	43 (35.3)	331 (26.2)	228 (33.3)	<.001	44 (31.2)	195 (31.0)	363 (27.9)	0.001	144 (34.9)	261 (36.8)	33 (14.9)	163 (22.7)	<.001
Central region	46 (37.7)	503 (39.8)	294 (42.9)		59 (41.8)	283 (44.8)	502 (38.6)		153 (37.1)	295 (41.6)	141 (63.5)	249 (34.7)	
South region	33 (27.1)	431 (34.1)	163 (23.8)		38 (27.0)	153 (24.3)	436 (33.5)		116 (28.1)	154 (21.7)	48 (21.6)	306 (42.6)	
**Urban vs rural residency**													
Urban	56 (45.9)	508 (40.2)	187 (27.3)	<.001	36 (25.5)	188 (29.8)	527 (40.5)	<.001	150 (36.3)	245 (34.5)	71 (32.0)	281 (39.1)	0.837
Rural	66 (54.1)	757 (59.8)	489 (72.7)		105 (74.5)	442 (70.2)	774 (59.5)		263 (63.7)	465 (65.5)	151 (68.0)	437 (60.9)	
**Wealth index**													
Poorest	22 (18.0)	193 (15.3)	122 (17.8)	<.001	25 (17.7)	127 (20.2)	185 (14.2)	<.001	71 (17.2)	125 (17.6)	47 (21.2)	93 (13.0)	0.001
Poorer	25 (20.5)	199 (15.7)	157 (22.9)		38 (27.0)	129 (20.5)	214 (16.5)		59 (14.3)	164 (23.1)	48 (21.6)	107 (14.9)	
Middle	22 (18.0)	248 (19.6)	160 (23.4)		26 (18.4)	145 (23.0)	259 (19.9)		92 (22.3)	137 (19.3)	49 (22.1)	152 (21.2)	
Richer	20 (16.4)	302 (23.9)	153 (22.3)		30 (21.3)	134 (21.3)	311 (23.9)		111 (26.9)	147 (20.7)	48 (21.6)	166 (23.1)	
Richest	33 (27.1)	323 (25.5)	93 (13.6)		22 (15.6)	95 (15.1)	335 (25.5)		137 (19.3)	137 (19.3)	30 (13.5)	200 (27.9)	
**Partner/Husband controlling behavior**													
No control	70 (57.4)	719 (56.8)	402 (58.7)	0.733	72 (51.1)	367 (58.3)	752 (57.8)	0.275	224 (54.2)	428 (60.3)	141 (63.5)	392 (54.6)	0.020
At least one type	52 (42.6)	546 (43.2)	283 (41.3)		69 (48.9)	263 (41.8)	549 (42.2)		189 (45.8)	282 (39.7)	81 (36.5)	326 (45.4)	
**IPV exposure**													
	96 (79.3)	964 (76.5)	536 (78.3)	0.563	107 (75.9)	487 (77.3)	1002 (77.3)	0.927	308 (74.9)	571 (80.7)	182 (82.0)	527 (73.5)	0.002
	25 (20.7)	297 (23.6)	149 (21.8)		34 (24.1)	143 (22.7)	294 (22.7)		103 (25.1)	137 (19.4)	40 (18.0)	190 (26.5)	

S1 Table B: Description of contraceptive practices by selected sociodemographic characteristics.

	Contraceptive practices												
	Current use of contraceptive methods				Length of use of contraceptives				Need for contraceptives				
	No use	Modern	Traditional	p-value	No use	2yrs or less	More than 2yrs	p-value	Unmet need	Met need	No want	No need	p-value
**Age**													
<19	134 (9.3)	29 (4.8)	1 (3.7)	<.001	134 (9.3)	29 (6.7)	1 (0.5)	<.001	35 (7.6)	30 (4.8)	95 (15.6)	4 (1.1)	<.001
20-29	598 (41.2)	247 (40.9)	8 (29.6)		598 (41.2)	200 (46.5)	51 (26.6)		198 (43.0)	255 (40.4)	325 (53.2)	70 (19.1)	
30-39	400 (27.5)	223 (36.9)	7 (25.9)		400 (27.5)	150 (34.9)	76 (39.6)		139 (30.2)	230 (36.5)	159 (26.0)	97 (26.5)	
40-49	318 (22.0)	105 (17.4)	11 (40.7)		318 (22.0)	51 (11.9)	64 (33.3)		89 (19.3)	116 (18.4)	32 (5.2)	195 (53.3)	
**Education[1]**													
No education	466 (32.3)	129 (21.4)	9 (33.3)	<.001	468 (32.3)	102 (23.7)	34 (17.7)	<.001	140 (30.4)	138 (21.9)	177 (29.0)	146 (39.9)	<.001
Primary	750 (52.1)	321 (53.2)	15 (55.6)		755 (52.1)	230 (53.5)	101 (52.6)		242 (52.5)	336 (53.3)	321 (52.5)	187 (51.1)	
Secondary and above	225 (15.6)	154 (25.5)	3 (11.1)		227 (15.7)	98 (22.8)	57 (29.7)		79 (17.1)	157 (24.9)	113 (18.5)	33 (9.0)	
**Currently employed**													
No	860 (59.7)	286 (47.4)	14 (51.9)	<.001	867 (59.8)	216 (50.2)	77 (40.1)	<.001	297 (64.4)	300 (47.5)	365 (59.7)	196 (53.6)	<.001
Yes	581 (40.3)	318 (52.7)	13 (48.2)		583 (40.2)	214 (49.8)	115 (59.9)		164 (35.6)	331 (52.5)	246 (40.3)	170 (46.5)	
**Live children**													
0	155 (10.7)	6 (1.0)	0	<.001	155 (10.7)	4 (0.9)	2 (1.0)	<.001	14 (3.0)	6 (1.0)	84 (13.8)	57 (15.6)	<.001
1-4	952 (66.1)	406 (67.2)	18 (66.7)		957 (66.0)	294 (68.4)	125 (65.1)		312 (67.7)	424 (67.2)	425 (69.6)	213 (58.2)	
5 or more	334 (23.2)	192 (31.8)	9 (33.3)		338 (23.3)	132 (30.7)	65 (33.9)		135 (29.3)	201 (31.9)	102 (16.7)	96 (26.2)	
**Regions**													
North region	452 (31.4)	138 (22.9)	12 (44.4)	<.001	456 (31.3)	109 (25.4)	39 (20.3)	<.001	124 (26.9)	150 (23.8)	196 (32.1)	132 (36.1)	<.001
Central region	650 (45.1)	191 (31.6)	2 (7.4)		605 (45.2)	144 (33.5)	44 (22.9)		211 (45.8)	193 (30.6)	296 (48.5)	140 (38.3)	
South region	339 (23.5)	275 (45.5)	13 (48.2)		341 (23.5)	177 (41.2)	109 (56.8)		126 (27.3)	288 (45.6)	119 (19.5)	94 (25.7)	
**Urban vs rural residency**													
Urban	467 (32.4)	273 (45.2)	11 (40.7)	<.001	470 (32.4)	172 (40.0)	109 (56.8)	<.001	150 (32.5)	284 (45.0)	193 (31.6)	124 (33.9)	<.001
Rural	974 (67.6)	331 (54.8)	16 (59.3)		980 (67.6)	258 (60.0)	83 (43.2)		311 (67.5)	347 (55.0)	418 (68.4)	242 (66.1)	
**Wealth index**													
Poorest	266 (18.5)	68 (11.3)	3 (11.1)	<.001	267 (18.4)	59 (13.7)	11 (5.7)	<.001	81 (17.6)	71 (11.3)	119 (19.5)	66 (18.0)	<.001
Poorer	306 (21.2)	68 (11.3)	7 (25.9)		309 (21.3)	61 (14.2)	11 (5.7)		99 (21.5)	75 (11.9)	133 (21.8)	71 (19.4)	
Middle	327 (22.7)	100 (16.6)	3 (11.1)		328 (22.6)	74 (17.2)	28 (14.6)		99 (21.5)	103 (16.3)	145 (23.7)	83 (22.7)	
Richer	311 (21.6)	158 (26.2)	6 (22.2)		313 (21.6)	111 (25.8)	51 (26.6)		99 (21.5)	164 (26.0)	125 (20.5)	87 (23.8)	
Richest	231 (16.0)	210 (34.8)	8 (29.6)		233 (16.1)	125 (29.1)	91 (47.4)		83 (18.0)	218 (34.6)	89 (14.6)	59 (16.1)	
**Partner/Husband controlling behavior**													
No control	860 (59.7)	320 (53.0)	11 (40.7)	0.004	862 (59.5)	230 (53.5)	99 (51.6)	0.020	268 (58.1)	331 (52.5)	371 (60.7)	219 (59.8)	0.018
At least one type	581 (40.3)	284 (47.0)	16 (59.3)		588 (40.6)	200 (46.5)	93 (48.4)		193 (41.9)	300 (47.5)	240 (39.3)	147 (40.2)	
**IPV exposure**													
No	1144 (79.6)	431 (71.6)	21 (77.8)	<.001	1152 (79.6)	307 (71.7)	137 (71.4)	<.001	363 (78.7)	452 (71.9)	489 (80.3)	290 (79.5)	0.002
Yes	294 (20.5)	171 (28.4)	6 (22.2)		295 (20.4)	121 (28.3)	55 (28.7)		98 (21.3)	177 (28.1)	120 (19.7)	75 (20.6)	
