# Supplementary material for: How women’s empowerment influences fertility-related outcomes and contraceptive practices: A cross-sectional study in Mozambique
Source: PLOS Glob Public Health. 2022 Sep 12;2(9):e0000670. doi: 10.1371/journal.pgph.0000670 (PMC10021614; doi:10.1371/journal.pgph.0000670)
Supplement: S2 Table — A: Crude and adjusted odds ratio (95% CI) from the multinomial logistic regression to estimate the association between the empowerment domains and fertility-related outcomes; and B: Crude and adjusted odds ratio (95% CI) from the multinomial logistic regression to estimate the association between the empowerment domains and contraceptive practices. (PDF) [file pgph.0000670.s002.pdf]

S2 Table A: Crude and adjusted odds ratio (95% CI) from the multinomial logistic regression to estimate the association between the empowerment domains and fertility-related outcomes.

	**Fertility-related outcomes, Crude and adjusted OR (95% CI)**													
	**Children ever born**<br>Ref: 5 or more				**Time interval between the last 2 births**<br>Reference: Less than 2 years				**Intentions for childbearing**<br>Ref: Less than 2 years					
	**Crude**		**Adjusted**		**Crude**		**Adjusted**		**Crude**			**Adjusted**		
	**0**	**1-4**	**0**	**1-4**	**2 to 6 years**	**1 or less children born in the previous 6 years**	**2 to 6 years**	**1 or less children born in the previous 6 years**	**2 years or more**	**Undecided**	**No want**	**2 years or more**	**Undecided**	**No want**
**Beliefs about violence**														
Low														
Middle	1.47<br>(0.90, 2.38)	**1.58<br>(1.26, 1.99)**	1.60<br>(0.95, 2.69)	**1.61<br>(1.22, 2.13)**	0.86<br>(0.53, 1.38)	0.93<br>(0.59, 1.47)	0.85<br>(0.53, 1.38)	0.82<br>(0.51, 1.32)	1.08<br>(0.80, 1.47	0.80<br>(0.53, 1.20)	0.86<br>(0.64, 1.16)	1.14<br>(0.82, 1.56)	0.87<br>(0.56, 1.34)	0.86<br>(0.62, 1.21)
High	1.25<br>(0.78, 2.02)	1.04<br>(0.82, 1.30)	1.27<br>(0.76, 2.12)	1.07<br>(0.82, 1.41)	0.88<br>(0.54, 1.43)	0.96<br>(0.60, 1.53)	0.88<br>(0.54, 1.43)	0.99<br>(0.61, 1.60)	0.91<br>(0.67, 1.24)	0.96<br>(0.64, 1.42)	**0.62<br>(0.45, 0.83)**	0.90<br>(0.65, 1.24)	0.95<br>(0.63, 1.43)	**0.59<br>(0.42, 0.82)**
**Decision-making**														
Low														
Middle	1.00<br>(0.62, 1.61)	1.14<br>(0.90, 1.44)	1.34<br>(0.80, 2.25)	**1.35<br>(1.02, 1.80)**	0.94<br>(0.59, 1.52)	1.13<br>(0.72, 1.79)	0.92<br>(0.57, 1.49)	0.96<br>(0.60, 1.53)	0.81<br>(0.60, 1.09)	1.09<br>(0.74, 1.61)	1.25<br>(0.93, 1.70)	0.90<br>(0.66, 1.23)	1.12<br>(0.74, 1.69)	1.18<br>(0.84, 1.66)
High	0.91<br>(0.57, 1.44)	1.02<br>(0.82, 1.28)	1.10<br>(0.66, 1.83)	1.04<br>(0.79, 1.38)	0.93<br>(0.59, 1.51)	1.32<br>(0.84, 2.08)	0.91<br>(0.56, 1.47)	1.13<br>(0.71, 1.83)	1.02<br>(0.76, 1.38)	0.93<br>(0.62, 1.40)	**1.75<br>(1.30, 2.37)**	1.16<br>(0.85, 1.59)	0.92<br>(0.60, 1.41)	**1.50<br>(1.06, 2.11)**
**Control over sexuality and safe sex**														
Low														
Middle	0.92<br>(0.59, 1.43)	**1.38<br>(1.11, 1.72)**	0.83<br>(0.51, 1.35)	1.19<br>(0.92, 1.55)	0.78<br>(0.50, 1.22)	0.96<br>(0.62, 1.48)	0.76<br>(0.48, 1.21)	0.86<br>(0.55, 1.35)	1.15<br>(0.87, 1.53)	0.90<br>(0.62, 1.31)	1.09<br>(0.83, 1.45)	1.19<br>(0.88, 1.60)	0.97<br>(0.66, 1.44)	1.08<br>(0.73, 1.48)
High	1.19<br>(0.72, 1.94)	**2.06<br>(1.61, 2.63)**	0.87<br>(0.50, 1.51)	**1.41<br>(1.04, 1.90)**	1.07<br>(0.64, 1.78)	1.11<br>(0.68, 1.81)	1.06<br>(0.62, 1.78)	0.96<br>(0.57, 1.62)	**1.62<br>(1.18, 2.22)**	1.04<br>(0.68, 1.59)	**1.40<br>(1.02, 1.92)**	**1.61<br>(1.15, 2.27)**	1.16<br>(0.73, 1.82)	1.42<br>(0.99, 1.05)

Adjusted for: number of live children, age, education, and region.

S2 Table B: Crude and adjusted odds ratio (95% CI) from the multinomial logistic regression to estimate the association between the empowerment domains and contraceptive practices.

	**Contraceptive practices, Crude and adjusted OR (95% CI)**											
	**Current use of contraceptives** Ref. No use				**Length of use of contraceptives** Ref. No use				**Need for contraceptives** Ref. Unmet need			
	**Crude**		**Adjusted**		**Crude**		**Adjusted**		**Crude**			**Adjusted**		
	**Modern**	**Traditional**	**Modern**	**Traditional**	**2 year or less**	**More than 2 years**	**2 year or less**	**More than 2 years**	**Met need**	**No want**	**No need**	**Met need**	**No want**	**No need**
**Beliefs about violence**												
Low (ref.)												
Middle	1.14 (0.91, 1.42)	0.91 (0.26, 3.17)	1.05 (0.82, 1.34)	0.89 (0.26, 3.13)	1.07 (0.83, 1.39)	1.27 (0.89, 1.81)	1.02 (0.78, 1.34)	1.12 (0.76, 1.64)	0.91 (0.68, 1.22)	**0.66 (0.49, 0.90)**	0.82 (0.59, 1.15)	0.83 (0.62, 1.12)	**0.64 (0.47, 0.87)**	0.79 (0.55, 1.15)
High	**0.78 (0.61, 0.99)**	**3.11 (1.13, 8.50)**	0.82 (0.63, 1.06)	**3.29 (1.20, 9.03)**	0.86 (0.66, 1.13)	0.79 (0.53, 1.18)	0.90 (0.68, 1.20)	0.89 (0.59, 1.35)	0.79 (0.58, 1.08)	0.92 (0.68, 1.24)	0.92 (0.65, 1.30)	0.83 (0.60, 1.12)	0.89 (0.65, 1.21)	0.92 (0.63, 1.33)
**Decision-making**												
Low (ref.)												
Middle	1.10 (0.86, 1.40)	0.39 (0.12, 1.26)	0.97 (0.75, 1.26)	0.35 (0.11, 1.13)	1.00 (0.77, 1.32)	1.21 (0.79, 1.84)	0.95 (0.71, 1.26)	0.91 (0.59, 1.42)	1.03 (0.74, 1.40)	0.75 (0.56, 1.01)	**1.42 (1.01, 2.00)**	1.12 (0.83, 1.50)	**0.74 (0.56, 0.97)**	0.95 (0.68, 1.34)
High	**1.48 (1.17, 1.87)**	1.29 (0.56, 2.96)	1.20 (0.93, 1.54)	1.11 (0.47, 2.61)	1.25 (0.96, 1.62)	**2.21 (1.51, 3.24)**	1.10 (0.84, 1.46)	1.48 (0.99, 2.23)	**1.37 (1.02, 1.83)**	0.82 (0.61, 1.10)	1.07 (0.76, 1.52)	**1.60 (1.12, 2.29)**	0.64 (0.48, 1.01)	0.94 (0.60, 1.46)
**Control over sexuality and safe sex**												
Low (ref.)												
Middle	**1.74 (1.37, 2.21)**	**3.61 (1.32, 9.85)**	**1.50 (1.16, 1.94)**	**3.60 (1.29, 9.99)**	**1.62 (1.24, 2.14)**	**2.57 (1.72, 3.84)**	**1.53 (1.15, 2.04)**	**1.82 (1.19, 2.79)**	**1.68 (1.26, 2.27)**	0.89 (0.67, 1.18)	0.96 (0.70, 1.31)	**1.47 (1.08, 1.99)**	0.87 (0.65, 1.16)	0.95 (0.67, 1.35)
High	**2.57 (2.01, 3.30)**	1.65 (0.46, 5.76)	**1.91 (1.46, 2.51)**	1.69 (0.47, 6.07)	**2.47 (1.87, 3.21)**	**2.95 (1.93, 4.51)**	**2.03 (1.50, 2.75)**	**1.82 (1.14, 2.88)**	**1.99 (1.46, 2.71)**	**0.71 (0.52, 0.97)**	**0.68 (0.47, 0.97)**	**1.55 (1.12, 2.15)**	**0.67 (0.48, 0.94)**	0.82 (0.55, 1.23)

Adjusted for: number of live children, age, education, region, and wealth quintile.
