# Supplementary figures and images for: How women’s empowerment influences fertility-related outcomes and contraceptive practices: A cross-sectional study in Mozambique
Source: PLOS Glob Public Health. 2022 Sep 12;2(9):e0000670. doi: 10.1371/journal.pgph.0000670 (PMC10021614; doi:10.1371/journal.pgph.0000670)

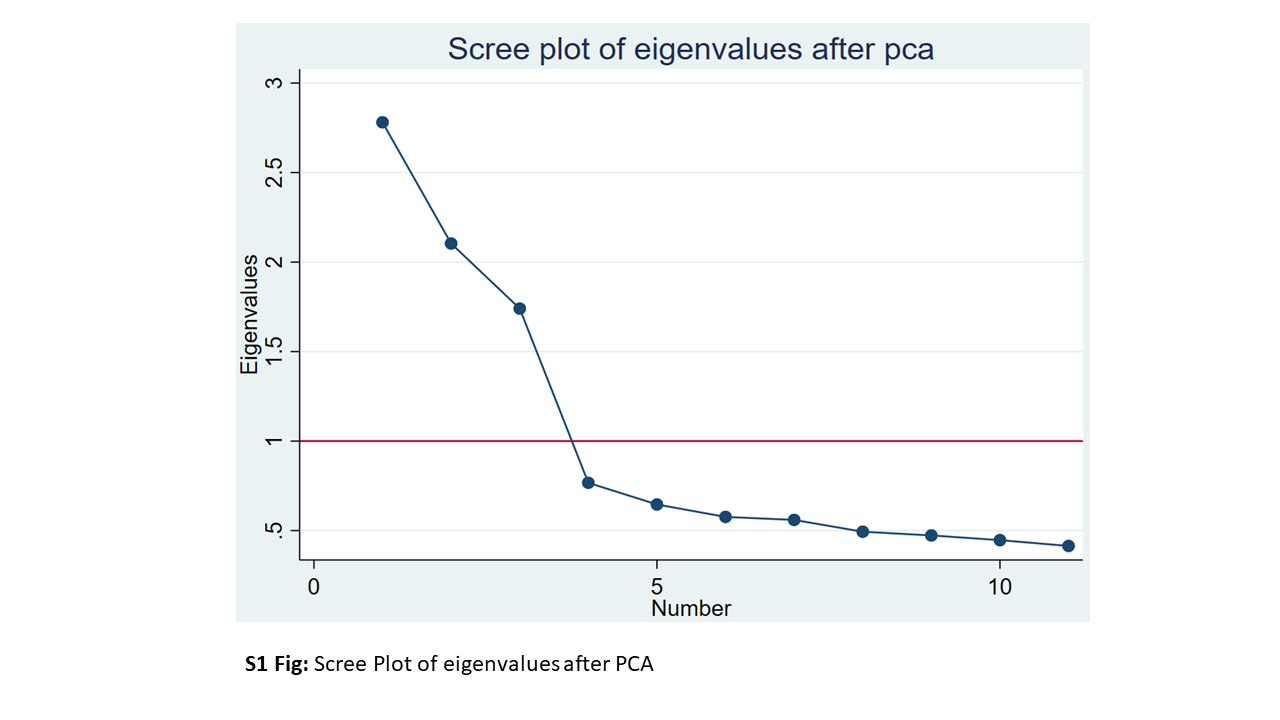

Supplement: S1 Fig — (TIF) [file pgph.0000670.s003.tif]
